# Microscale Interfacial Polymerization on a Chip

**DOI:** 10.1002/anie.202110974

**Published:** 2021-10-05

**Authors:** Marco Rocca, Maxime Dufresne, Marie Salva, Christof M. Niemeyer, Emmanuel Delamarche

**Affiliations:** ^1^ IBM Research Europe—Zurich Säumerstrasse 4 CH-8803 Rüschlikon Zurich Switzerland; ^2^ Institute of Biological Interfaces (IBG1) Karlsruhe Institute of Technology Hermann-von-Helmholtz-Platz 1 76344 Eggenstein-Leopoldshafen Germany

**Keywords:** capillary-driven flow, hydrogels, interfaces, microfluidics, polymerization

## Abstract

Forming hydrogels with precise geometries is challenging and mostly done using photopolymerization, which involves toxic chemicals, rinsing steps, solvents, and bulky optical equipment. Here, we introduce a new method for in situ formation of hydrogels with a well‐defined geometry in a sealed microfluidic chip by interfacial polymerization. The geometry of the hydrogel is programmed by microfluidic design using capillary pinning structures and bringing into contact solutions containing hydrogel precursors from vicinal channels. The characteristics of the hydrogel (mesh size, molecular weight cut‐off) can be readily adjusted. This method is compatible with capillary‐driven microfluidics, fast, uses small volumes of reagents and samples, and does not require specific laboratory equipment. Our approach creates opportunities for filtration, hydrogel functionalization, and hydrogel‐based assays, as exemplified by a rapid, compact competitive immunoassay that does not require a rinsing step.

## Graphical abstract

PEG‐based hydrogels can be precisely localized in capillary‐driven microfluidic chips by in situ interfacial polymerization. This allows using hydrogels for filtration, studying diffusion processes of solutes across the hydrogels and rapid bioassays.

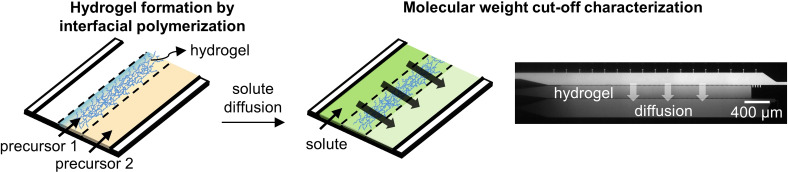

Hydrogels are excellent materials for microfluidic applications in biomedicine and biosensing owing to their compatibility with proteins and numerous solvents, permeability to small chemicals, transparency, potential for functionalization, and programmable stiffness.[^1^, ^2^, ^3^, ^4^, ^5^] For example, hydrogels have been employed for fabricating membranes in chips for microdialysis,[^6^, ^7^] filtration,^[8]^ preconcentration of proteins in electrophoresis,^[9]^ or studying solvophoresis^[10]^ and diffusophoresis.^[11]^ Likewise, hydrogels are frequently applied for the immobilization of biomolecular receptors in microfluidics to enable sensitive detection of proteins,[^12^, ^13^] nucleic acids,^[14]^ or small‐molecule targets.^[15]^ Moreover, due to their tunable response to temperature, pH, light, electric and magnetic fields, hydrogels are increasingly being used as microfluidic actuators, which leads to numerous novel applications.[^16^, ^17^, ^18^] However, controlling the geometry of a hydrogel at the microscale remains a challenge and techniques currently used to form hydrogels locally in microfluidics mainly employ photopolymerization[^19^, ^20^] or local confinement.[^21^, ^22^] Since these approaches often require toxic chemicals, such as photoinitiators, and/or bulky and expensive equipment to control polymerization conditions, there is a clear need for a simple, ready‐to‐use platform that allows convenient preparation and characterization of biocompatible hydrogels with a well‐defined microscale geometry. To address this demand, we report here on the use of sealed capillary‐driven microfluidic chips for the in situ production of biocompatible hydrogels by taking advantage of interfacial polymerization.[^23^, ^24^, ^25^] Using polyethylene‐glycol (PEG)‐based hydrogels as model systems, we demonstrate that our platform enables rapid prototyping of materials with respect to their polymerization properties and solute diffusion profiles.

The general approach to in situ formation of PEG hydrogels by interfacial polymerization in a microfluidic chip is shown in Figure 1. We used sealed capillary‐driven silicon microfluidic chips, which have well‐defined dimensions and flow properties and can be manufactured in large numbers.^[26]^ The chips were designed to contain three channels separated by capillary pinning structures, which define the interface between the two precursor solutions (Figure 1 a). The width of the capillary pinning structures and the gap between them both measure 10 μm. Further details about the microfluidic chip are given in Figure S1 and in section II in the Supporting Information. Furthermore, a capillary pump and a hydraulic resistance were designed to ensure a constant and fast enough flow rate to make diffusion‐driven concentration gradients across the sample channel negligible. As model system for hydrogel formation, we used the fast and robust polymerization method based on thiol‐maleimide coupling,[^27^, ^28^] which can be performed under physiological conditions. Moreover, PEG hydrogels were chosen because of their adjustable chemical composition and mechanical properties, protein biocompatibility and ease of chemical functionalization.^[29]^ In our method, two precursors in aqueous solutions, 4‐armed PEG maleimide (4PM) and PEG‐dithiol (PDT) (Figure 1 b), are sequentially introduced into the central and donor channel, respectively, of the microfluidic chip and the capillary pinning structures define the interface between the two solutions (Figure 1 c). The diffusion of PDT through the 4PM solution results in the formation of the hydrogel in the central channel. A third channel, dubbed as sample channel, can be used to provide a sample of interest containing solutes, which can interact with, or diffuse through, the formed hydrogel. A detailed description of the workflow used to form the hydrogel on chip is provided in Figure S2 and Supporting Information, Movies M1 and M2.

**Figure 1:**
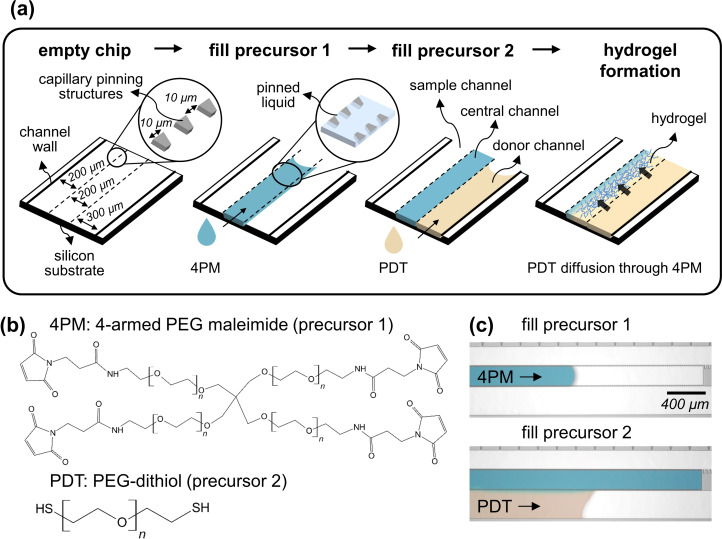
Formation of a PEG hydrogel in situ by interfacial polymerization inside a microfluidic chip. a) The hydrogel precursors (4PM and PDT) are introduced sequentially into parallel microchannels, which are delimited by capillary pinning structures and walls. PDT diffuses quickly through 4PM and the hydrogel forms only in the central channel. b) Chemical structure of the precursors. c) Optical micrographs showing the filling of 4PM in the central channel with pinning of the liquid preventing its excursion to the adjacent channels, followed with filling of the PDT precursor in the donor channel. A blue dye was added to the 4PM solution for better visualization. The contrast and color of the pictures were enhanced digitally to improve readability.

After filling the chip with the two precursors, the PDT diffusion front was observed while propagating through the 4PM‐filled central channel within ca. 2 min (Figure 2). In fact, owing to a difference in the refractive index between the two precursor solutions,^[30]^ the advancing diffusion front of PDT was visible as a propagating line by using bright field microscopy (Figure 2 a). The propagation of the PDT front over time followed a square root function, as expected for a diffusion of PDT through 4PM (Figure 2 b). To confirm that the observed propagating line correlates with the diffusion of PDT, a control experiment was performed using a pH‐sensitive fluorescent reporter dye in the 4PM solution (Figure S3) to indicate the occurrence of the rapid thiol‐maleimide reaction in a basic reaction environment.^[27]^ An increase in fluorescence intensity was observed as a moving front overlapping with the propagating line visible in bright field microcopy. Hence, the simple detection of the propagating line can be used as a near real‐time indicator of hydrogel formation in the microfluidic chip. Although not required for the studies shown below, unreacted PDT solution in the donor channel could be easily removed if needed by equipping the chip with a valve,[^31^, ^32^] to release remaining polymer precursors (e.g., PDT) downstream and flush the donor channel with a desired solution.

**Figure 2:**
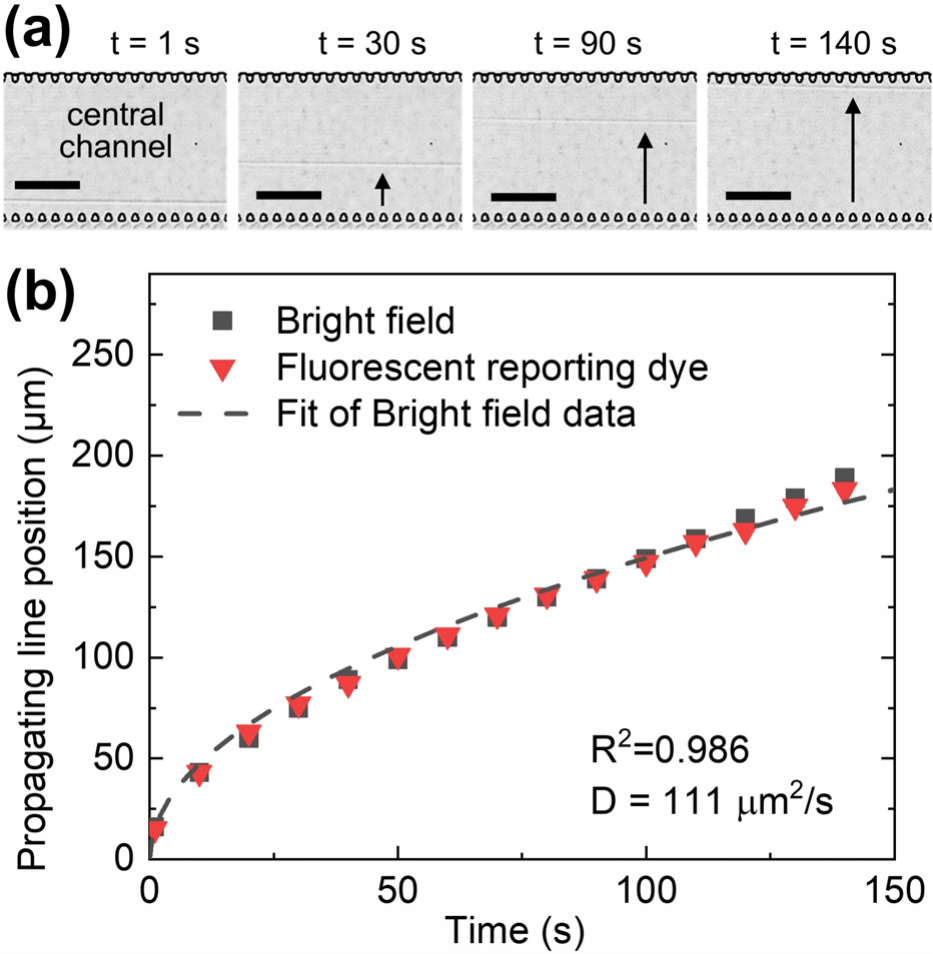
Visualization of the PDT diffusion front across the central channel filled with 4PM. a) Bright field micrographs of the central channel taken at four time intervals after the filling of the donor channel (not shown here). The diffusion front of PDT manifests as a propagating line and is highlighted by the arrow. Scale bars: 100 μm. b) Graph showing the position of the propagating line across the central channel as a function of time (black squares) and where experimental data points are fitted using a square root function (dashed line), yielding a diffusion coefficient of 111 μm^2^ s^−1^. Additionally, a pH sensitive dye was added to the 4PM solution to visualize the propagating front of PDT through 4PM (red triangles). The corresponding fluorescence images are shown in Figure S3a.

The in situ formation of a microstructured hydrogel with a well‐defined interface can be useful for microfluidic applications requiring filtration or localization of analytes and reaction intermediates. For such applications, it is important to assess the filtration characteristics and molecular weight cut‐off (MWCO) of the formed hydrogels for representative molecules that can be supplied in the sample channel. This was done using a constant flow of fluorescent molecules of variable molecular weight in water (Figure 3; Supporting Information, Figure S4 a,b, Movie M3). Diffusion of these molecules into the hydrogel was followed in real time using fluorescence microscopy (Figure S5). The graphs in Figure 3 clearly indicate that solutes of low molecular weight (Rhodamine 479 Da, TRITC‐dextran 4.4 kDa) diffuse quickly through the hydrogel, while solutes with a larger molecular weight reveal much slower diffusion (TRITC‐dextran 20 kDa) or nearly none (TRITC‐dextran 155 kDa). A control experiment with hydrolyzed, non‐polymerizable 4PM in the central channel, showed as expected that indeed only the formation of a hydrogel leads to the observed filtration of the solute (Figure S4c, d).

**Figure 3:**
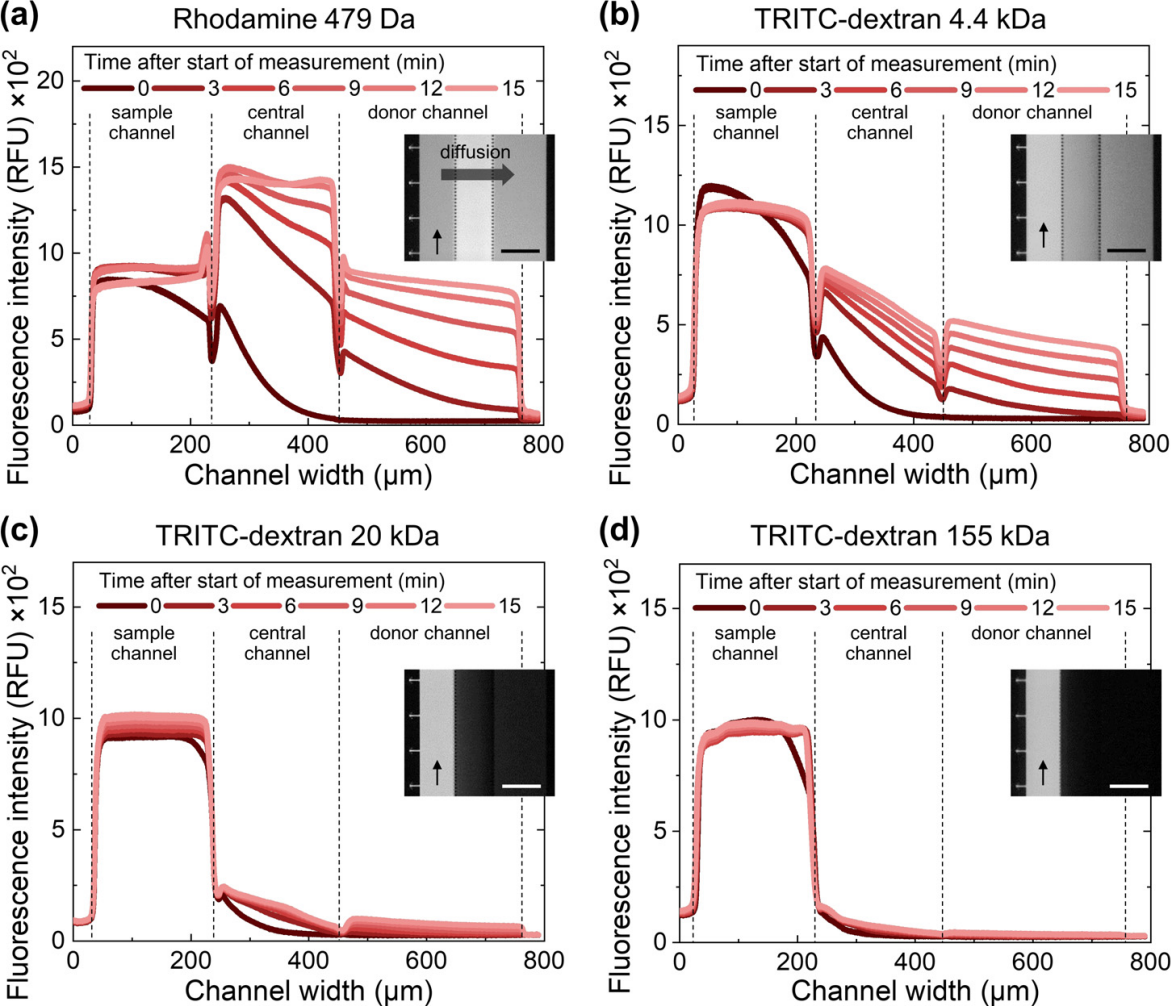
Diffusion profiles over time of fluorescent molecules having different molecular weights a) 479 Da, b) 4.4 kDa, c) 20 kDa, and d) 155 kDa) and that are introduced in a sample channel. Depending on their molecular weights, molecules may cross the hydrogel in the central channel and reach the donor channel. Insets show fluorescence microscope images of the three channels taken 20 minutes after introducing the fluorescent molecules in the sample channel. The scalebars in the inset represent 200 μm and the vertical arrow the direction of flow when introducing the solutions containing the fluorescent molecules in the sample channels. The small fluctuations in the fluorescence intensity at the interface between adjacent channels visible in (a) and (b) are due to the presence of capillary pinning structures (see detailed explanations in Figure S6). For additional graphs using 10 and 70 kDa solutes and polymerization controls, see Figure S4. Representative images of these experiments are shown in Figure S5.

To increase the usefulness of our platform for preparing hydrogel materials and studying or controlling solute diffusion profiles, we attempted to change the MWCO of three hydrogels by altering their mesh size (ξ). This was done by changing the concentration of the PDT and 4PM precursors and the length of the PEG chains in 4PM (Figure 4). Specifically, we doubled the volume fraction of the hydrogel to 20 % to obtain a smaller mesh size or used 4PM 10 kDa instead of 2 kDa to form a larger mesh size. Based on these conditions for hydrogel formation, we then computed the mesh sizes of the three hydrogels using a theoretical model for ideal PEG hydrogel networks[^33^, ^34^] and obtained mesh sizes of 5.3 and 12.6 nm for hydrogels formed as indicated above, and 6.7 nm for the hydrogel used in Figures 2 and 3. We again used hydrolyzed 4PM in the central channel as a control experiment. We then defined the diffusion index of fluorescent solutes through these hydrogels as the ratio between the fluorescence intensity in channels on either side of the hydrogel (i.e. donor channel and sample channel), which was measured 20 minutes after loading the sample solution. This index indicates how much analytes are able to diffuse through a hydrogel having a specific MWCO.

**Figure 4:**
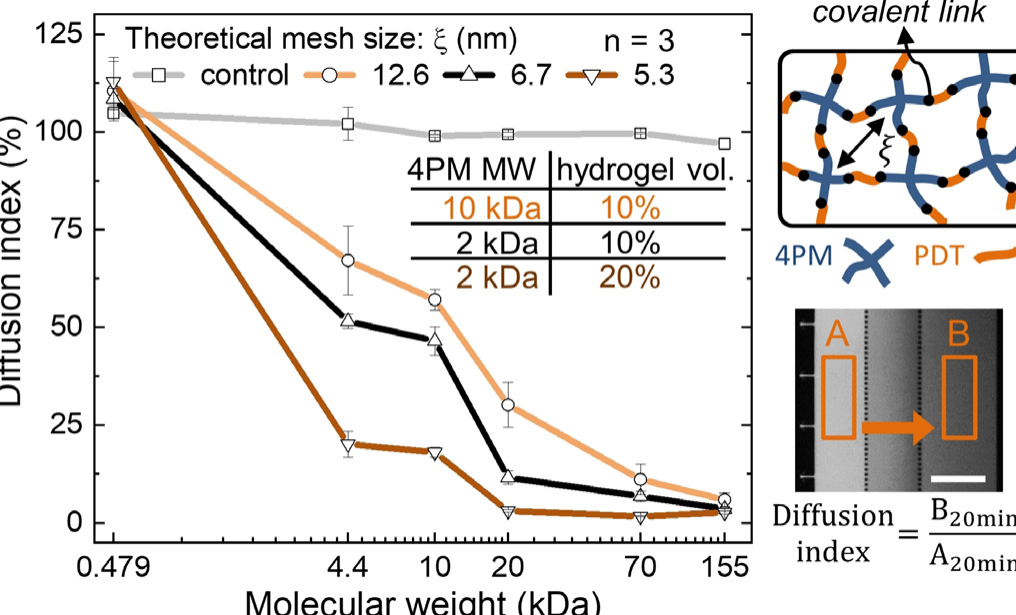
Characterization of the diffusion of TRITC dyes conjugated to dextrans of various molecular weights through hydrogels having different mesh sizes. The diffusion index is defined as the ratio of dyes in the donor channel and sample channel after 20 min. Theoretical mesh size (*ξ*) and preparation of the hydrogels: i) yellow line, *ξ*=12.6 nm, 10 mM 4PM 10 kDa and 20 mM PDT ii) black line, *ξ*=6.7 nm, 35 mM 4PM 2 kDa and 70 mM PDT iii) brown line, *ξ*=5.3 nm, 70 mM 4PM 2 kDa and 140 mM PDT, and iv) gray line, control (hydrolyzed 4PM). The fluorescence microscope image shows the regions of interest where fluorescence intensities were used to calculate the diffusion index of TRITC‐dextran (4.4 kDa) through a hydrogel with a theoretical mesh size of 6.7 nm. Scalebar: 200 μm. Raw data for the curves related to 5.3 and 12.6 nm mesh sizes are shown in Figure S7 and S8.

Figure 4 shows that Rhodamine (479 Da) diffused rapidly into the donor channel, leading to a very high diffusion index. Solutes with a MW of already 4.4 kDa revealed a diffusion index ranging from 67 % down to 20 % depending on the hydrogel characteristics. As expected, analytes with a large MW of 70 and 155 kDa hardly diffused through the hydrogel, resulting in a diffusion index that tends to zero regardless of hydrogel composition. In addition, the observed diffusion indices correlated with the theoretical mesh size, where the hydrogel with the largest mesh size (12.6 nm) was the most permeable to analytes. In contrast, the control experiments with hydrolyzed 4PM did not show significant filtration capability for the solutes. These results show that the MWCO of the hydrogels can easily be modified by changing the hydrogel characteristics and quantified by testing its permeability to molecules with different MWs. In addition, using monodisperse solutes, it should be possible to precisely determine experimentally diffusion characteristics of solutes and the mesh size of hydrogels using a simple experimental setup as shown here. Thus, in situ polymerized hydrogels could be used to study the monodispersity and purity of recombinant proteins at very low sample volumes (Figure S9).

To further illustrate the utility of in situ polymerized hydrogels to implement bioassays, we realized a novel format of a rapid competitive immunoassay for small‐molecule analytes, using biotin as a model analyte in a proof‐of‐concept. Since our approach can easily produce hydrogels from more than two components, we produced a biofunctional ternary hydrogel by incorporating antibodies directed against biotin directly into the hydrogel during 4PM/PDT polymerization. A solution containing a mixture of the target analyte as well as a fluorescently labeled derivative of the analyte was then introduced in the sample channel (Figure 5 a). The analyte competes with the labelled‐analyte for the antibody binding sites inside the hydrogel, thus affecting the fluorescence profile of the accumulated labelled‐analyte in the hydrogel (Figure 5 b). The variation of the fluorescence profile across the hydrogel correlates with the concentration of analyte over more than 3 orders or magnitude (Figure 5 c). A control experiment performed with hydrogel‐trapped antibodies that are not specific for the target analyte confirmed that the accumulation of fluorescence is due to specific antibody‐analyte interactions (Figure S10). This assay can be performed in just 6 minutes and does not require a rinsing step, which is commonly performed for surface‐based competitive immunoassays.^[35]^ Moreover, our competitive hydrogel assay could be particularly useful for measuring protein‐free small‐molecule analytes by filtering out protein‐bound analytes using the appropriate hydrogel mesh size.

**Figure 5:**
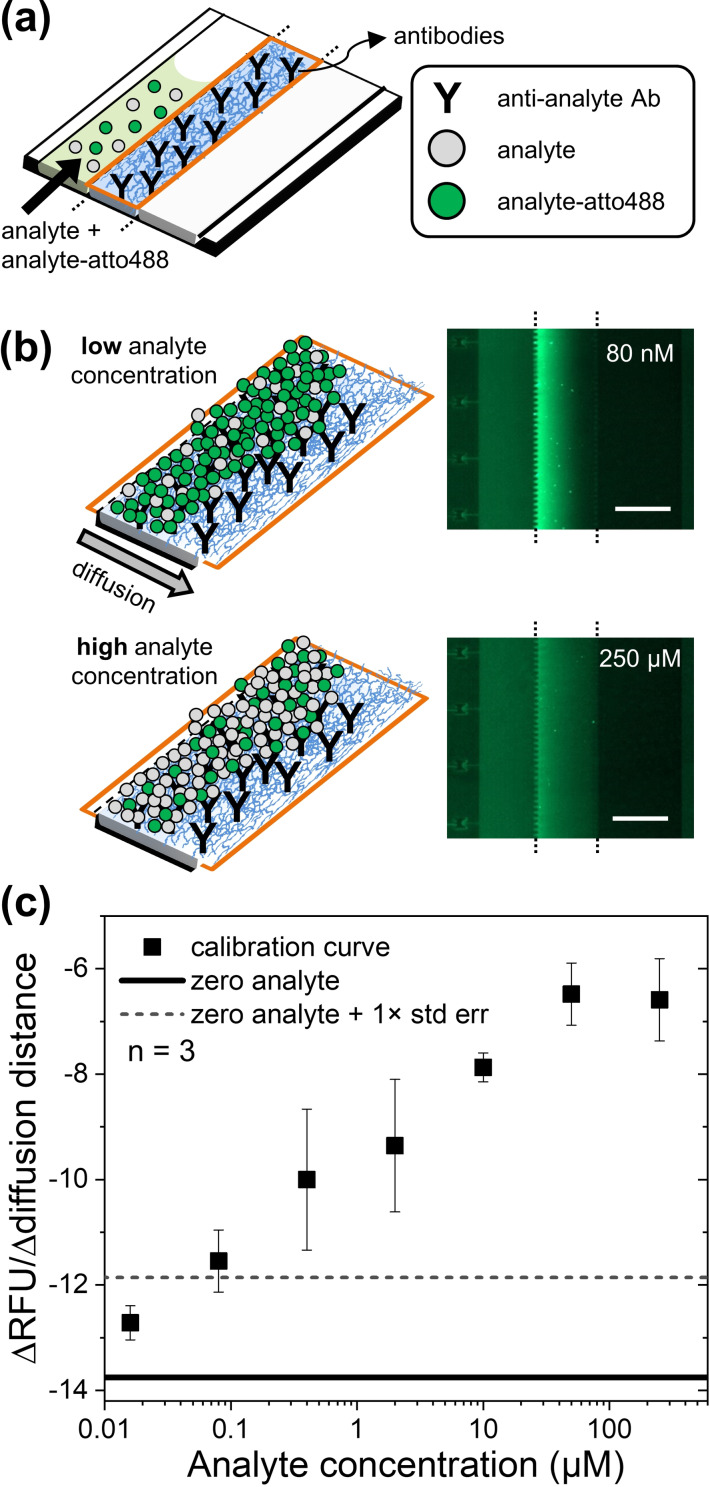
Competitive immunoassay in a hydrogel. a) Anti‐analyte antibodies are trapped in a hydrogel and 3 μL of a solution containing an analyte and a labelled analyte (labelled with atto488) are loaded into the sample channel. b) Competitive immunoassay and corresponding fluorescence images of the sample‐, central‐ and donor‐channel taken 6 minutes after loading a solution containing 200 nM analyte‐atto488 and two representative concentrations of analyte (80 nM and 250 μM) in the sample channel. Scalebars 200 μm. c) Calibration curve of the competitive assay (analyte concentrations: 16 nM up to 250 μM). The data points show the mean value of the slope of the fluorescence profile across the hydrogel 6 minutes after loading the sample solution in the sample channel. The solid line corresponds to a negative control (zero analyte concentration and 200 nM analyte‐atto488) and the dashed line to the zero analyte plus one standard error of the mean. The error bars indicate the standard error of the mean.

Our assay is also superior to standard sandwich assays, which would require a detection antibody to migrate into the hydrogel, resulting in mass transport‐limited binding and/or unwanted enrichment within the hydrogel and associated poor signal‐to‐background ratios. In contrast, in our assay, the antibody is used as a capture binding site that is permanently located in the hydrogel. Smaller capture reagents, e.g., affibodies with a MW of 6 kDa, could also be used in our method, for example, by being previously linked to a polymerizable group and then linked to the hydrogel during polymerization. Since hydrogels have very low non‐specific interactions with analytes and signal‐reporting molecules, non‐specific accumulation of interfering species in the hydrogel compartment is unlikely to occur.

In summary, we reported on a novel method enabling the rapid preparation of precisely localized hydrogels and their characterization in terms of diffusion properties for various solutes. The formation of such hydrogels inside a microfluidic chip only requires about 200 nL of precursor solutions, allowing economical use of reagents, can be achieved in less than 3 minutes, and can follow any geometry delimited by capillary pinning structures. In addition, this method is compatible with both pressure‐driven and capillary‐driven flows. Thereby, it does not require specific pumping peripherals, can be kept simple and portable, and should also be compatible with centrifugal microfluidic platforms, which are nowadays gaining importance for point‐of‐care diagnostics applications. Our method is versatile and could be employed for several applications, including size‐selective filtration and localized assays. Immobilization of receptors in a hydrogel precisely localized in a microfluidic device can lead to accumulation of analytes in a convenient‐to‐read signal area as we demonstrated by performing a rapid competitive immunoassay on chip. By combining filtration and assay localization, our method could also facilitate the detection of analytes, which exist in polymeric forms, such as the inflammatory biomarker C‐reactive protein. A similar possibility exists for numerous analytes and drugs for which the protein‐free concentration needs to be determined. This work therefore opens exciting opportunities in the area of biosensing and protein characterization where hydrogels are often used.

## Conflict of interest

The authors declare no conflict of interest.

## Supporting information

As a service to our authors and readers, this journal provides supporting information supplied by the authors. Such materials are peer reviewed and may be re‐organized for online delivery, but are not copy‐edited or typeset. Technical support issues arising from supporting information (other than missing files) should be addressed to the authors.

Supporting Information
