# Supplementary material for: Microscale Interfacial Polymerization on a Chip
Source: Angew Chem Int Ed Engl. 2021 Oct 5;60(45):24064–9. doi: 10.1002/anie.202110974 (PMC8597160; doi:10.1002/anie.202110974)
Supplement: Supplementary file 1 — Supporting Information [file ANIE-60-24064-s001.pdf]

Angewandte Chemie
Eine Zeitschrift der Gesellschaft Deutscher Chemiker

Supporting Information

Microscale Interfacial Polymerization on a Chip

Marco Rocca, Maxime Dufresne, Marie Salva, Christof M. Niemeyer, and Emmanuel Delamarche**

anie_202110974_sm_miscellaneous_information.pdf

Table of Contents

Experimental Procedures... 2
Supporting Figures 4
Author Contributions... 13
References 13

Experimental Procedures

I. Reagents

All reagents were purchased from Sigma-Aldrich unless otherwise indicated. 4PM 2 kDa, 4PM 10 kDa and PDT were purchased from CreativePEGWorks (Chapel Hill, NC, USA). TRITC-dextran 10 kDa and TRITC-dextran 70 kDa were purchased from ThermoFisher Scientific (Waltham, MA, USA).

II. Fabrication of microfluidic chips

The microfluidic chips were designed using L-edit (Mentor Graphics, Oregon, USA) and patterned on a soda lime mask using a direct laser writer (DWL 2000 Heidelberg-Instruments, Heidelberg, Germany). The channel walls and capillary pinning structures of the microfluidic chips were fabricated on a 4” silicon wafer (Si-Mat, Germany) using one photolithographic step of 15 µm SU-8 3010 (MicroChem Corp., Massachusetts, USA). A thin photoresist (AZ-4562) was spin-coated on top of the microstructures to protect them from debris during the dicing process. The photoresist was removed using acetone and the chips were rinsed with isopropyl alcohol. The chips were sealed using a dry film resist (DF-1050, EMS Inc., USA). The dry film resist was exposed by a collimated 365 nm UV light (Thorlab MCWHL5-C5, 80 mW, 43 mm beam diameter, 700 mA, 4.4 V, 5.51 mW/cm^2) for 1 min through a photomask. The dry film resist was crosslinked only above the sample-, central-, and donor- channels in order to prevent deformation of the dry film resist due to the swelling of the hydrogel. The photomask was printed on a transparent projector sheet with a Canon Bubble Jet i9100 Printer. The contact area between the sample and the hydrogel should be maximized to avoid obstructing the diffusion of sample into the hydrogel. Hence, the gap between the capillary pinning structures should be as large as possible and the size of the capillary pinning structures should be as small as possible. With the current fabrication process, the gap between the capillary pinning structures needs to be smaller than 15 µm to ensure a reliable pinning of the 4PM solution. To include a safety margin, we used a gap of 10 µm (Figure S1). The size of the capillary pinning structures is limited by the resolution of the photolithographic process, which for our case requires the capillary pinning structures to be at least 10 µm wide. The resulting pitch of the capillary pinning structures pattern is 20 µm.

III. Preparation of precursor solutions and fluorescent samples

4PM 2 kDa was dissolved in DMSO at a concentration of 350 mM and then diluted to 35 mM (for ξ = 6.7 nm) or 70 mM (for ξ = 5.3 nm) in MilliQ water containing 200 mM triethanolamine (final concentration). 4PM 10 kDa was dissolved in water at a concentration of 65 mM and then diluted to 10 mM (for ξ = 12.6 nm) in MilliQ water containing 200 mM triethanolamine (final concentration). PDT was dissolved in MilliQ water at a concentration of 700 mM and then diluted to 70 mM (for ξ = 6.7 nm), 140 mM (for ξ = 5.3 nm) or 20 mM (for ξ = 12.6 nm) in MilliQ water containing 200 mM triethanolamine (final concentration). Fluorescent samples were prepared in MilliQ water with the following concentrations: Rhodamine B and TRITC-dextran 10 kDa = 5 mg/mL; TRITC-dextran 4.4 kDa, TRITC-dextran 20 kDa, TRITC-dextran 70 kDa, TRITC-dextran 155 kDa = 10 mg/mL.

IV. Hydrogel formation on chip

0.2 µL of 4PM were loaded in pad 1. After the central channel filled (10 s), the excess 4PM solution in pad 1 was dried with an absorbing paper in order to avoid a pressure difference between the liquids in pad 1 and pad 2 once PDT is loaded. This pressure difference would cause a capillary flow between the two pads and create inhomogeneities in the hydrogel. Similarly, 0.2 µL of PDT were loaded in pad 2. After the donor channel filled (5 s), the excess PDT solution was dried with an absorbing paper. 2 min 30 s were necessary for the PDT to diffuse through 4PM and form a hydrogel in the central channel.

V. Diffusion experiment using fluorescent molecules

The microfluidic chips were placed in a Petri dish having a 4 cm^2 wet paper to form a humidity chamber (Figure S2). 3 µL of sample were loaded in pad 3 and then the lid of the plastic box was closed. The humidity chamber was placed under the microscope and the measurement was started. The capillary pump provides a reliable pressure difference to pull a sample solution from the loading pad into the chip and we used a hydraulic resistance (e.g. microchannel with a narrow width) to control the flow rate of the sample through the sample channel. The hydraulic resistance was designed to provide a constant flow rate of sample in the sample channel for at least 15 minutes and

at the same time to ensure a sufficiently high flow rate to make sample concentration gradients due to diffusion negligible compared to the sample channel width[1].

VI. Competitive assay in a hydrogel

Anti-analyte antibodies were mixed with 4PM before forming a hydrogel. The final concentration of anti-analyte antibodies in the hydrogel was 1 mg/mL. Solutions containing analyte and analyte-atto488 were prepared with a constant concentration of analyte-atto488 (200 nM) and varying concentrations of analyte (16 nM, 80 nM, 400 nM, 2 µM, 10 µM, 50 µM, 250 µM and a zero analyte as control). 3 µL of sample were loaded in pad 3 following the same procedure described in the paragraph above. Fluorescence images were taken every minute for 20 minutes using a microscope.

VII. Equipment

Fluorescence images were recorded using a Nikon Eclipse 90i microscope, a 10× objective (Plan Apo Nikon, NA = 0.45) and a DS-1QM/H CCD camera (Nikon). Snapshots and videos of the chips were taken using a Leica MZ16 stereomicroscope equipped with a Nikon J1 digital camera, a custom-made microscope equipped with an 8-megapixel CMOS camera and controlled by a Raspberry Pi (https://github.com/IBM/MicroscoPy) and a smartphone camera (Samsung Galaxy S7, SM-G930F).

VIII. Data processing

Images were analyzed using ImageJ and the graphs plotted using Origin 2018.

Supporting Figures

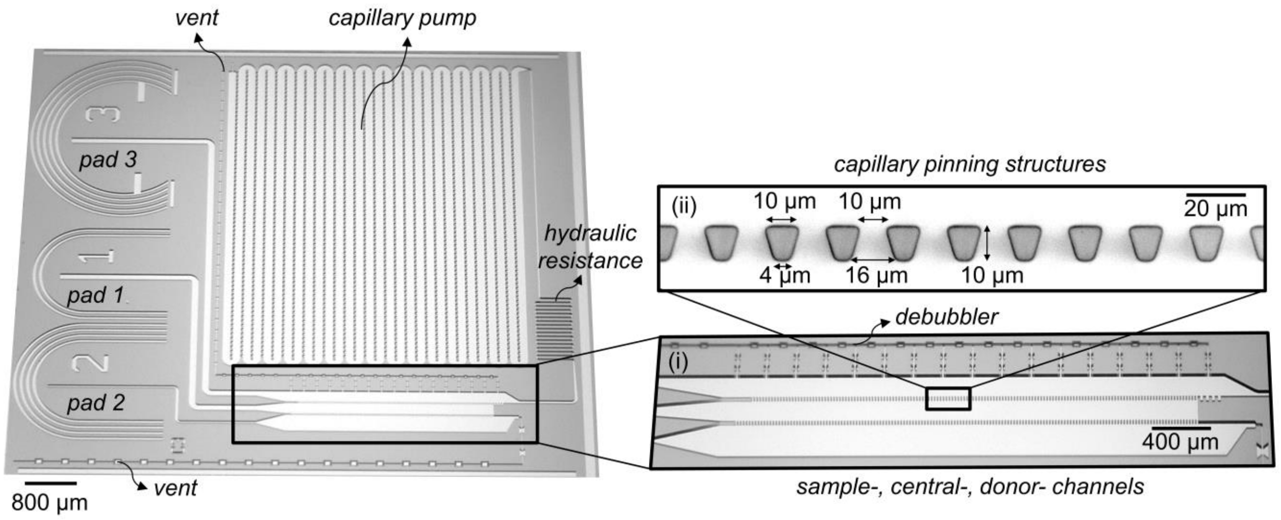

Figure S1. Photographs of a capillary-driven microfluidic chip before sealing it with a dry film resist. Pads 1 to 3 are used to load 4PM, PDT and a sample, respectively. Vents allow air displaced by the filling liquids to exit the flow paths. A hydraulic resistance is used to set the desired flow rate of the sample and a capillary pump provides a constant flow rate of sample for at least 15 minutes. (i) Additional vents are placed in the sample channel to avoid formation of air bubbles while the sample fills the channel (“debubbler”). (ii) Capillary pinning structures used to pin 4PM.

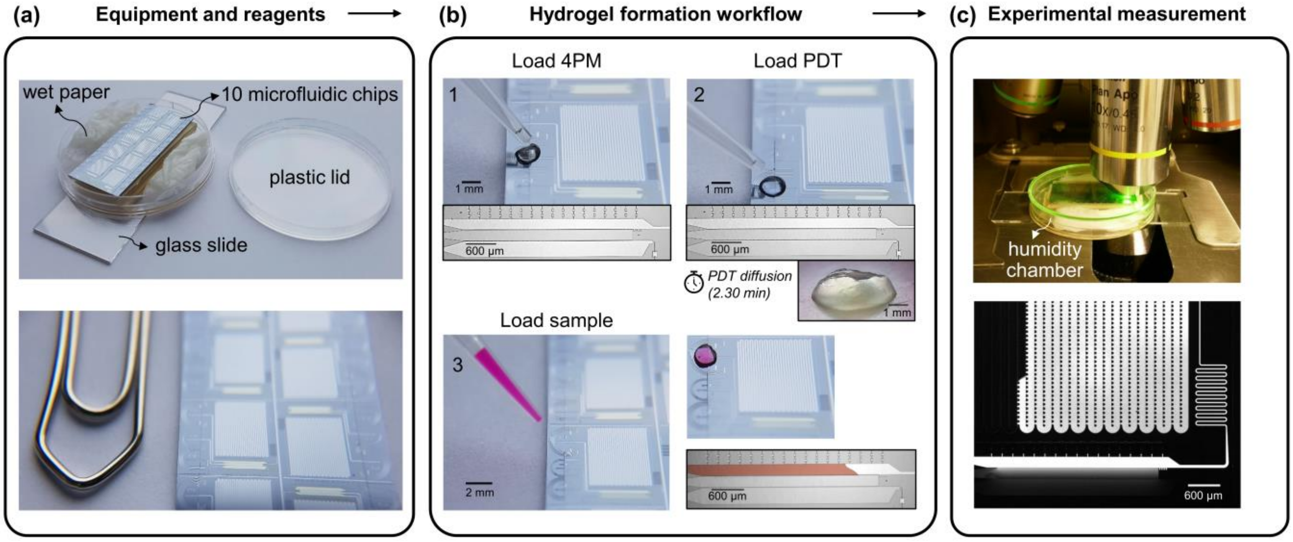

Figure S2. Experimental setup and workflow for the formation of a hydrogel. (a) A stripe containing 10 microfluidic chips is placed into a plastic box (Petri dish) with a lid. A wet paper is added into the box to keep a high relative humidity and prevent evaporation of the drop of sample added to the chip. This prevents variation for the sample concentration due to evaporation during the experiment. (b) Workflow for the formation of the hydrogel. First, 0.2 µL of 4PM are loaded into pad 1. After the central channel is filled, the drop of 4PM is dried with an absorbing paper. 0.2 µL of PDT are loaded into pad 2. After the donor channel is filled, the drop of PDT is dried with an absorbing paper. PDT takes about 2 min and 30 s to diffuse through 4PM and form a hydrogel. Next, 3 µL of sample are loaded into pad 3. (c) The measurements of the diffusion of the fluorescent dyes through the hydrogel are performed using an epifluorescence microscope.

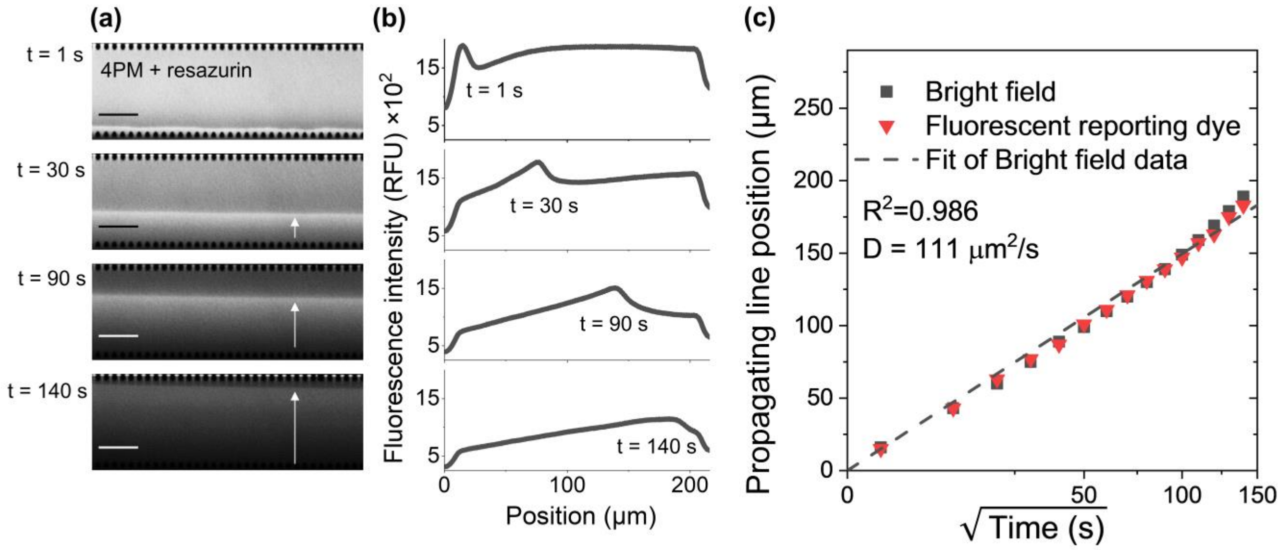

Figure S3. Visualization of the PDT diffusion front through the central channel. (a) A pH sensitive fluorescent reporting dye (1 mM resazurin) was added to the 4PM solution. The higher pH of the diffusing PDT solution causes resazurin to emit a stronger fluorescence intensity. Since the change in fluorescence is transient (moving peak), we speculate that the basic thiolates generated during polymerization briefly and locally shift the pH upward during polymerization. Scalebar: 100 µm. (b) Fluorescence intensity across the central channel at four different timeframes. The maximum in the fluorescence intensity propagates across the central channel, which is represented in (c) as the distance of propagation of the diffusion front as a function of the square root of time.

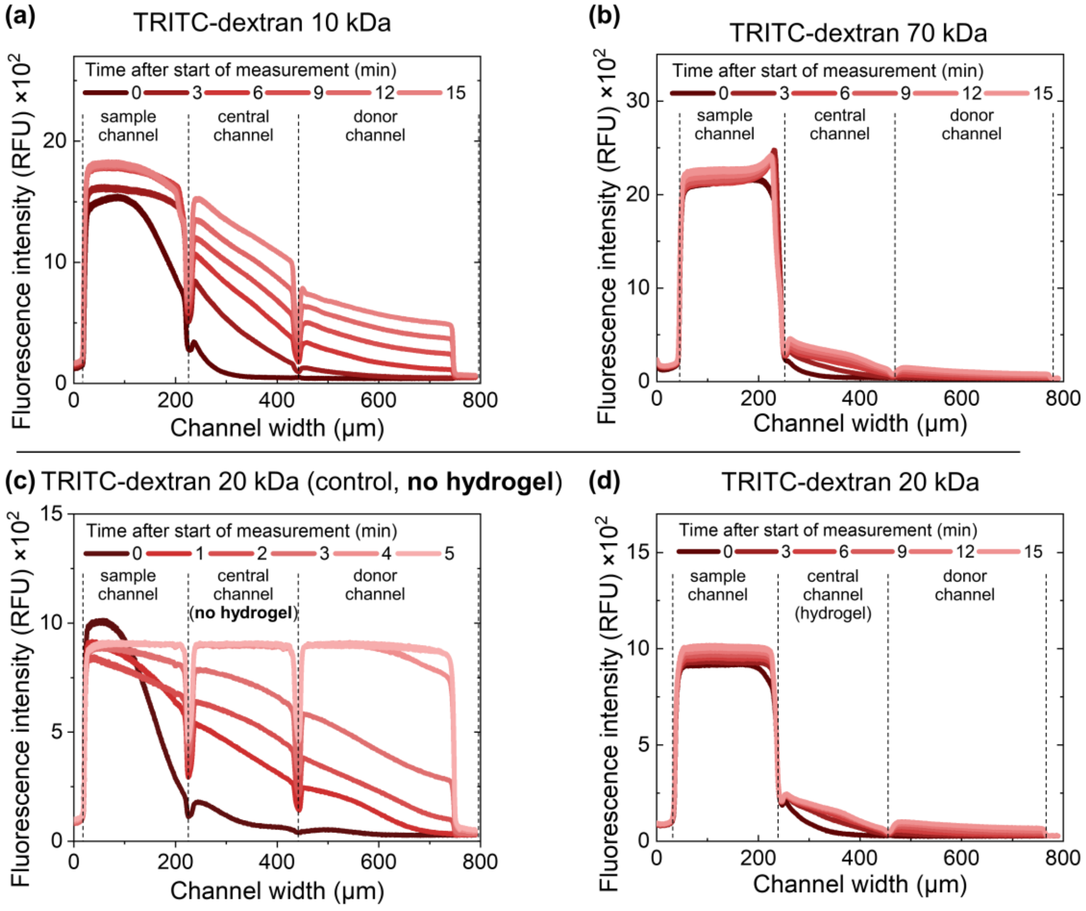

Figure S4. Characterization of the diffusion profile of different samples through the central channel. Fluorescence intensity profiles of (a) TRITC-dextran 10 kDa and (b) TRITC-dextran 70 kDa. (c) Control experiment: Fluorescence intensity profile of TRITC-dextran 20 kDa. For this experiment, hydrolyzed 4PM was inserted into the central channel instead of fresh 4PM and hence the hydrogel did not form. (d) The fluorescence profile of TRITC-dextran 20 kDa across the hydrogel formed using fresh 4PM is provided for comparison. Note that the control experiment in (c) was performed by introducing hydrolyzed 4PM in the central channel. Specifically, 4PM was hydrolyzed in an aqueous solution for 24 h at room temperature. In this case, and unlike in Figure S2b, no hydrogel was formed by pipetting PDT on a drop of the hydrolyzed 4PM standing on a glass slide. With hydrolyzed 4PM loaded in the central channel, TRITC-dextran 20 kDa supplied in the sample channel reached the donor channel within few minutes, which clearly indicates that diffusion of molecules across the central channel is modulated by a hydrogel network.

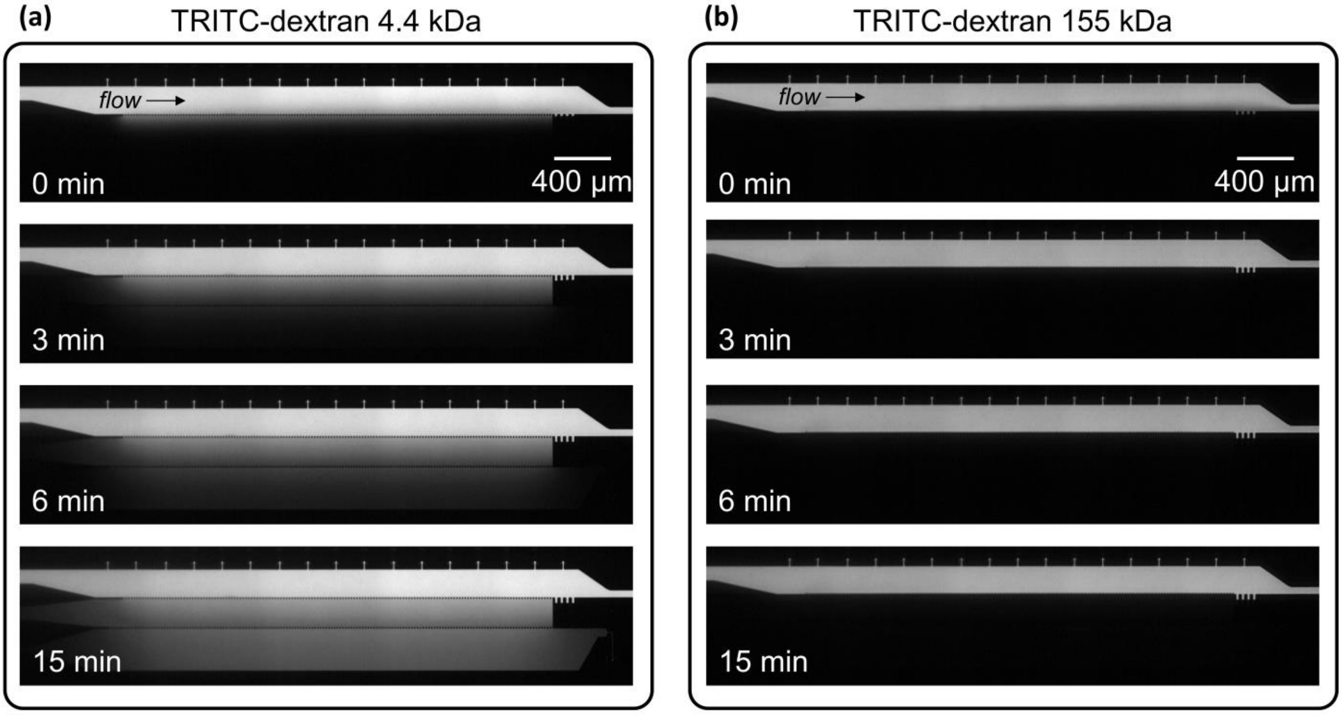

Figure S5. Fluorescence images of the sample-, central-, and donor-channels at different times during a diffusion experiment. The theoretical mesh size of the hydrogel formed here is 6.7 nm and the molecular weight of the sample is (a) 4.4 kDa and (b) 155 kDa. The flow direction of the sample solution is indicated in the sample channel at t = 0 min.

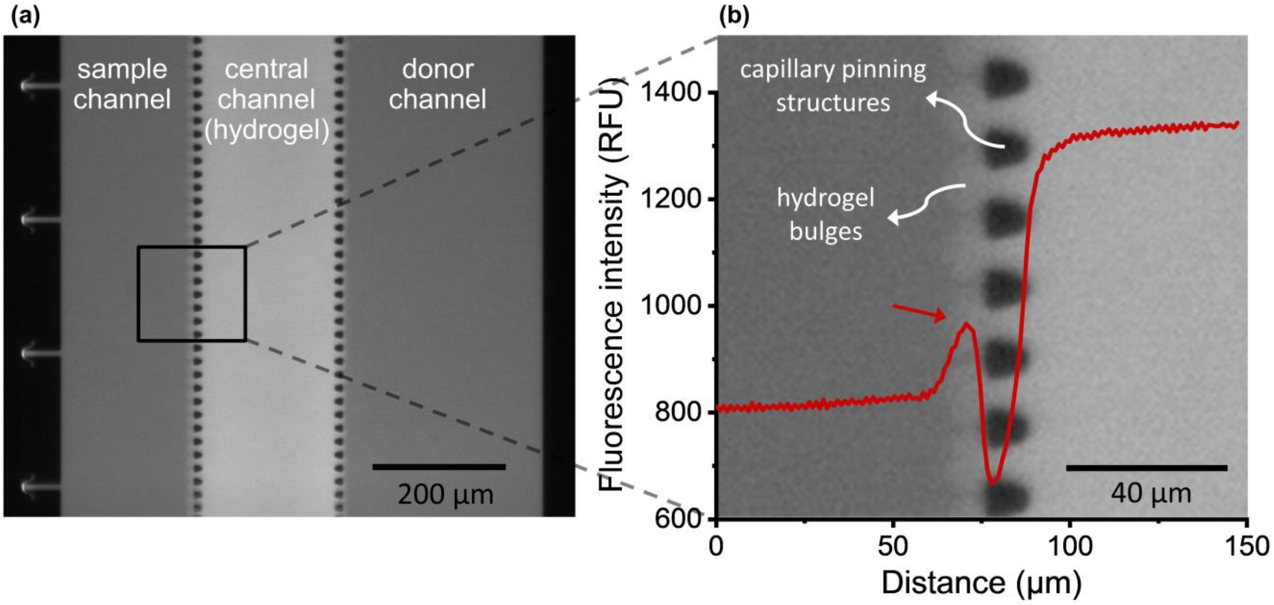

Figure S6. Fluorescence image of the sample-, central-, and donor-channels taken 15 minutes after a sample containing Rhodamine was loaded in the sample channel. (a) Full image and (b) magnified view of the interface between the sample- and central-channel. The red curve shows the fluorescence intensity across the channels averaged in the direction parallel to the channels. Note the local fluorescence maximum (red arrow) that occurs due to the small hydrogel bulges, which extend toward the sample channel. In addition, the local fluorescence minimum is due to the capillary pinning structures, which contribute to a lower average fluorescence intensity at the interface between the sample channel and the hydrogel.

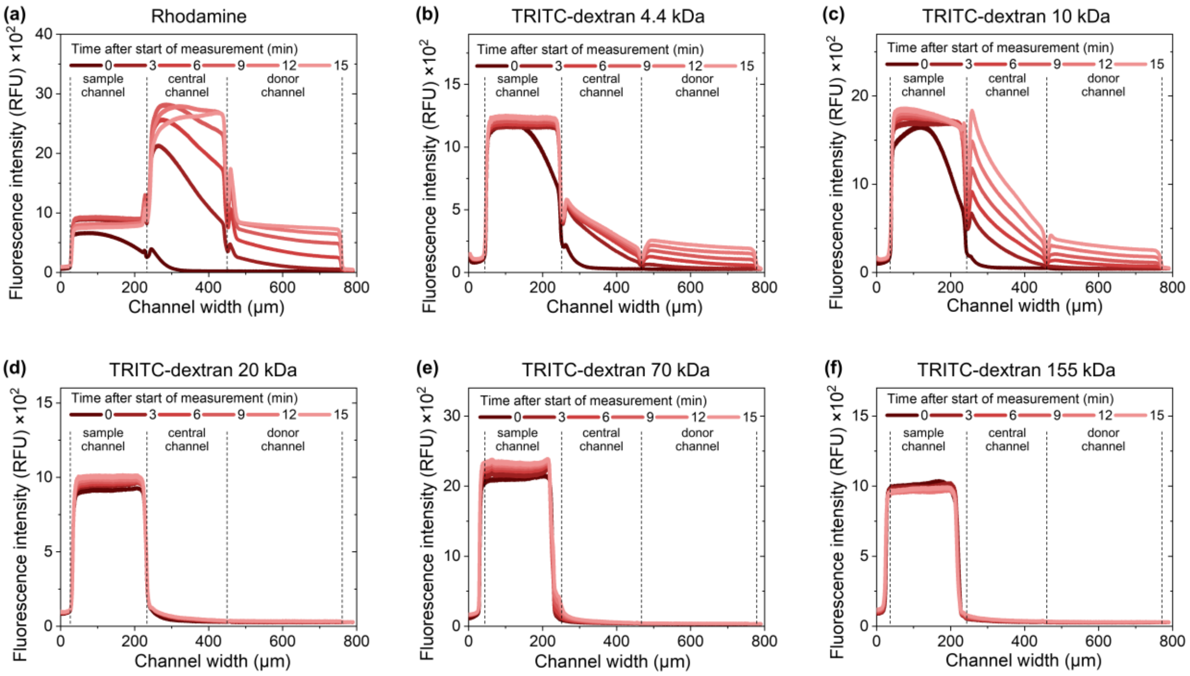

Figure S7. Diffusion profiles over time of fluorescent molecules having different molecular weights ((a) Rhodamine 479 Da, (b) TRITC-dextran 4.4 kDa, (c) TRITC-dextran 10 kDa, (d) TRITC-dextran 20 kDa, (e) TRITC-dextran 70 kDa, (f) TRITC-dextran 155 kDa) through a hydrogel with a theoretical mesh size of 5.3 nm.

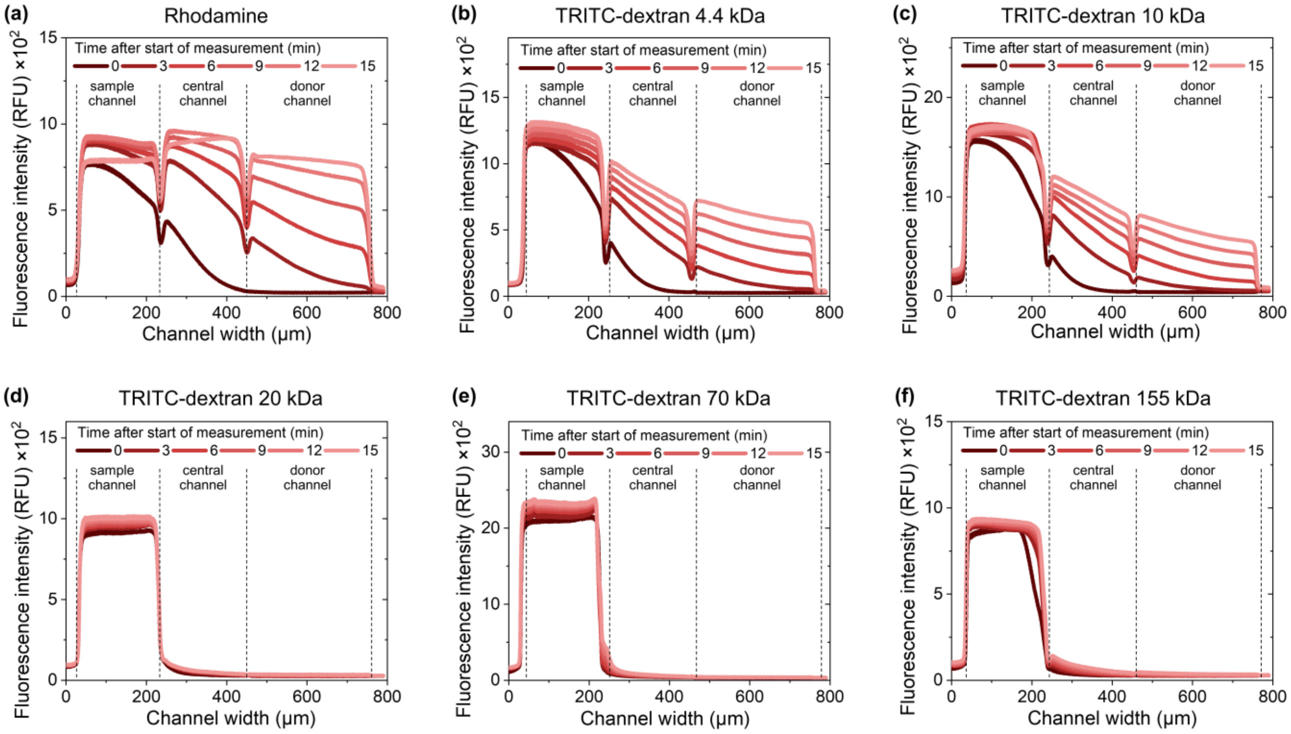

Figure S8. Diffusion profiles over time of fluorescent molecules having different molecular weights ((a) Rhodamine 479 Da, (b) TRITC-dextran 4.4 kDa, (c) TRITC-dextran 10 kDa, (d) TRITC-dextran 20 kDa, (e) TRITC-dextran 70 kDa, (f) TRITC-dextran 155 kDa) through a hydrogel with a theoretical mesh size of 12.6 nm.

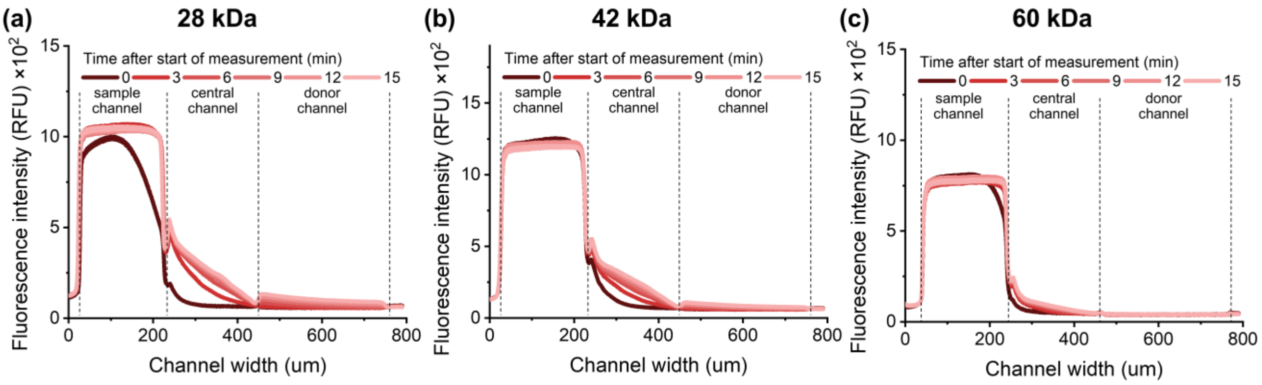

Figure S9. Diffusion profiles over time of red fluorescent proteins having different molecular weights ((a) 28 kDa, (b) 42 kDa, (c) 60 kDa) through a hydrogel with a theoretical mesh size of 6.7 nm.

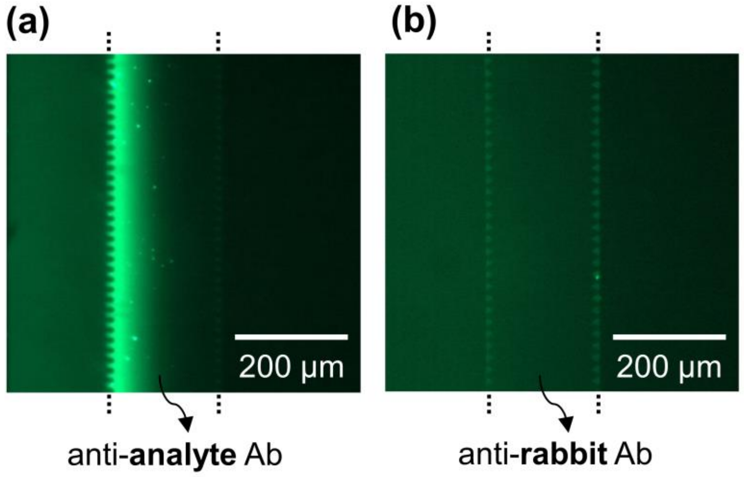

Figure S10. Fluorescence images of the sample (left), central (middle), and donor (right) channels, where (a) anti-analyte and (b) anti-rabbit (negative control) antibodies were trapped in the hydrogel (central channel). The images were taken 6 minutes after loading a solution containing 200 nM analyte-atto488 in the sample channel. The free diffusion of analyte-atto488 through the hydrogel in (b) demonstrates that the accumulation of fluorescence signal in the hydrogel in (a) occurs through a specific analyte-antibody interaction.

Author Contributions

M.R., C.N., and E.D. conceived the research, M.R. designed and fabricated the microfluidic chips, M.R. and M.D. performed the experiments. M.D. and M.S. implemented a theoretical model to guide the experiments, M.R. and M.S. analyzed the data. M.R., C.N., and E.D interpreted the data. C.N. and E.D. supervised the work. All the authors contributed to the writing of the manuscript.

References

[1] A. Olanrewaju, M. Beaugrand, M. Yafia, D. Juncker, *Lab Chip* **2018**, *18*, 2323–2347.
